# Effectiveness of 2024/25 KP.2 Vaccine Against Outpatient COVID‐19 in Canada

**DOI:** 10.1111/irv.70222

**Published:** 2026-03-05

**Authors:** Lea Separovic, Suzana Sabaiduc, Yuping Zhan, Samantha E. Kaweski, Sara Carazo, Romy Olsha, Richard G. Mather, Christine Lacroix, Maan Hasso, Inès Levade, Isabelle Meunier, Agatha N. Jassem, Katie Dover, Ruimin Gao, Nathalie Bastien, Danuta M. Skowronski

**Affiliations:** ^1^ British Columbia Centre for Disease Control Vancouver Canada; ^2^ Institut National de Santé Publique du Québec Québec Canada; ^3^ Public Health Ontario Toronto Canada; ^4^ Queen's University Kingston Canada; ^5^ National Microbiology Laboratory, Public Health Agency of Canada Winnipeg Canada; ^6^ University of British Columbia Vancouver Canada

**Keywords:** COVID‐19, public health, respiratory illness, vaccine effectiveness, vaccines

## Abstract

The Canadian Sentinel Practitioner Surveillance Network used the test‐negative design to assess KP.2 vaccine effectiveness (VE) against medically attended outpatient COVID‐19 between November 2024 and April 2025 among participants ≥ 12 years. Analyses included 5410 controls (19% vaccinated) and 435 COVID‐19 cases (10% vaccinated). Nearly three‐quarters (72%) of contributing case viruses were genetically characterized, with XEC (35%) and KP.3.1.1 (38%) variants most commonly identified. Vaccination reduced COVID‐19 risk by approximately half (54%; 95% CI: 36–68) relative to unvaccinated individuals. VE was greatest during the first 2 months postvaccination, reducing risk by two‐thirds and declining to minimal protection by the fifth month postvaccination.

## 1 Background

Prior to the 2024/25 respiratory season, SARS‐CoV‐2 circulation in the United States and Canada peaked between August and September 2024, followed by lower level circulation compared to previous seasons [1]. Whereas 2024/25 vaccine strains elsewhere (e.g., Europe, United States [2, 3]) included JN.1 or KP.2, in Canada, a universal fall vaccination campaign offered publicly funded mRNA vaccines containing KP.2 only. Ultimately, circulating viruses were mostly comprised of other JN.1 descendant or recombinant strains such as KP.3, XEC and their sublineages [1]. Few published estimates of 2024/25 COVID‐19 vaccine effectiveness (VE) are available to date, and most have only reported interim season findings. Here, the Canadian Sentinel Practitioner Surveillance Network (SPSN) estimates end‐of‐season 2024/25 KP.2 VE against outpatient COVID‐19, including genetic characterization of contributing case viruses and exploration of waning effects.

## 2 Methods

We estimated COVID‐19 VE by test‐negative design among patients presenting to community‐based sentinel practitioners in the provinces of British Columbia (bc), Ontario and Quebec within 7 days of onset of acute respiratory illness (ARI) inclusive of new or worsening cough potentially due to infection. Respiratory specimens were tested by accredited provincial laboratories using molecular detection assays. Fall KP.2 vaccination began in participating provinces in late‐September to mid‐October, 2024. We include specimens collected between epi‐weeks 44–18 (27 October 2024 to 03 May 2025). Ethical review waivers were provided by respective ethics committees in participating provinces.

In primary analyses, vaccine status was based upon information from provincial immunization registries (PIR), as previously [4]. In supplementary analyses, we compare VE based on self‐report. We exclude children < 12 years of age (due to more complex dosing recommendations), influenza‐positive controls (to address correlated vaccination behaviours), and those vaccinated < 2 weeks before onset or with unknown vaccine status or timing [5]. Logistic regression estimated odds ratios (OR) comparing the likelihood of vaccination among test‐positive cases versus test‐negative controls, with adjustment for age group, province, and calendar time. VE was derived as (1‐OR)*100%. We sought whole genome sequencing of all case viruses (GISAID Epi Set ID: EPI_SET_250903wr), assigning SARS‐CoV‐2 lineages using Pango nomenclature (Table S1). We assessed VE by variant and time since vaccination (TSV) as feasible.

## 3 Results

Participant characteristics are detailed in Table 1. Cases were slightly older than controls (median ages 49 vs. 46 years). The proportion vaccinated among controls (19%) was comparable to the most recently available 2023/24 fall campaign vaccine coverage estimates in Canada (19% among those ≥ 5 years) [7].

**TABLE 1:** Participant profile, COVID‐19 vaccine effectiveness analysis, Canadian Sentinel Practitioner Surveillance Network (SPSN), 27 October 2024 to 03 May 2025 (Weeks 44–18).

Characteristics	All ARI participants (column %)							Proportion COVID‐19 vaccinated^a^ (row %)						
	Overall		COVID‐19 cases		COVID‐19 controls		*p* ^b^	Overall		*p* ^c^	COVID‐19 cases		COVID‐19 controls	
	*n*	%	*n*	%	*n*	%		*n*	%		*n*	%	*n*	%
N (row %)	5845	100	435	7	5410	93	NA	1097	19	NA	44	10	1053	19
**Age group (years)**														
12–49	3207	55	223	51	2984	55	0.292	264	8	< 0.001	7	3	257	9
50–64	1278	22	103	24	1175	22		241	19		4	4	237	20
≥ 65	1360	23	109	25	1251	23		592	44		33	30	559	45
Median (IQR)	47 (33–63)		49 (36–65)		46 (33–63)		0.002	66 (64–79)		< 0.001	72 (64–79)		66 (50–75)	
**Sex**														
Female	3744	64	278	64	3466	64	0.889	732	20	0.046	28	10	704	20
Male	2073	35	156	36	1917	35		361	17		15	10	346	18
Unknown	28	0	1	0	27	0	NA	4	14	NA	1	100	3	11
**Comorbidity** ^**d**^														
No	3839	66	288	66	3551	66	0.5	520	14	< 0.001	25	9	495	14
Yes	1580	27	127	29	1453	27		457	29		18	14	439	30
Unknown	426	7	20	5	406	8	NA	120	28	NA	1	5	119	29
**Province**														
British Columbia	1452	25	49	11	1403	26	< 0.001	354	24	< 0.001	11	22	343	24
Ontario	2671	46	277	64	2394	44		423	16		28	10	395	16
Quebec	1722	29	109	25	1613	30		320	19		5	5	315	20
**Epi‐week of specimen collection, 2024/25** ^e^														
44–45	408	7	34	8	374	7	< 0.001	6	1	< 0.001	0	0	6	2
46–47	415	7	34	8	381	7		28	7		2	6	26	7
48–49	451	8	30	7	421	8		60	13		3	10	57	14
50–51	542	9	47	11	495	9		92	17		2	4	90	18
52–1	439	8	43	10	396	7		95	22		5	12	90	23
2–3	619	11	58	13	561	10		132	21		5	9	127	23
4–5	544	9	51	12	493	9		113	21		3	6	110	22
6–7	536	9	62	14	474	9		107	20		9	15	98	21
8–9	509	9	22	5	487	9		112	22		5	23	107	22
10–11	399	7	18	4	381	7		89	22		1	6	88	23
12–13	332	6	10	2	322	6		90	27		2	20	88	27
14–15	290	5	13	3	277	5		75	26		2	15	73	26
16–18	361	6	13	3	348	6		98	27		5	38	93	27
**Received COVID‐19 vaccine from April to September in 2024** ^f^														
Yes	247	4	17	4	230	4	0.765	168	68	< 0.001	11	65	157	68
No	5598	96	418	96	5180	96		929	18		33	9	896	19

*Note:* Unless otherwise specified, values displayed in the columns represent the number of specimens per category and percentages are relative to the total.

Abbreviations: ARI, acute respiratory illness; IQR, interquartile range; NA, not applicable.

^a^ Vaccination status refers to vaccination as part of fall 2024/25 vaccine campaigns, based on provincial immunization registry. Participants vaccinated < 2 weeks before onset of symptoms or with unknown vaccination status or timing were excluded. No participants in VE analyses received spring 2025 booster vaccine prior to symptom onset.

^b^ *p*‐values for comparison between cases and controls were derived by two‐way chi‐squared test or Wilcoxon rank‐sum test.

^c^ *p*‐values for comparison between vaccinated and not vaccinated were derived by two‐way chi‐squared test or Wilcoxon rank‐sum test. The number not vaccinated can be derived by subtracting the number vaccinated from the total ARI participants, by row.

^d^ Includes chronic comorbidities that place individuals at higher risk of serious complications from influenza as defined by Canada's National Advisory Committee on Immunization [6].

^e^ Missing specimen collection dates were imputed as the date the specimen was received and processed at the laboratory minus 2 days.

^f^ Participants vaccinated between 1 April 2024 and 30 September 2024. Earliest start date for fall 2024/25 vaccine campaigns in SPSN provinces was 30 September 2024 for high‐risk groups in Ontario; however, all vaccinated SPSN participants received fall dose on or after 1 October 2024.

Percent SARS‐CoV‐2 positivity among SPSN specimens remained low and stable at ~10% between epi‐weeks 44–7, declining to ≤ 5% most weeks thereafter (Figure S1). We successfully sequenced 312/435 (72%) case viruses, with a consistent mix of multiple JN.1 sublineages and recombinants, with no single dominant escape variant identified (Figure S2). Over one‐third were XEC (35%; 110/312) or KP.3.1.1 (38%; 118/312, including descendant MC). XEC contribution successively increased between epi‐weeks 44–46 (21%; 7/34), 47–49 (32%; 12/37) and 50–52 (49%; 21/43), remaining ≤ 50% thereafter. KP.3.1.1 contribution decreased during the same respective periods (71% (24/34), 54% (20/37) and 37% (16/43)), with equal proportions of ancestral KP.3.1.1 (52%; 31/60) and descendant MC (48%; 29/60) across epi‐weeks 44–52, but with lower KP.3.1.1 (18%; 9/51) than MC (80%; 41/51) between epi‐weeks 1–9. The emerging LP.8.1 variant comprised just 12% (37/312) of sequenced viruses overall, reaching 33% (9/27) during epi‐weeks 10–18 but with low case detection overall during that period.

**FIGURE 1:**
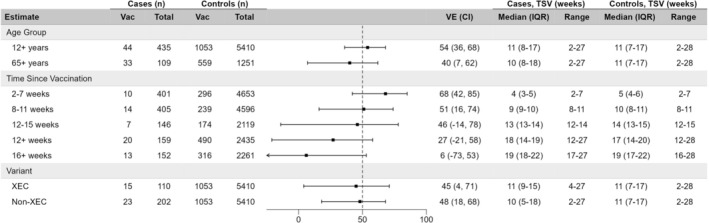
Vaccine effectiveness against acute respiratory illness due to SARS‐CoV‐2, Canadian Sentinel Practitioner Surveillance Network (SPSN), 27 October 2024 to 03 May 2025 (epi‐weeks 44–18) (*n* = 5845). Abbreviations: CI, confidence interval; IQR, interquartile range; TSV, time since vaccination; Vac, vaccinated; VE, vaccine effectiveness. Odds ratios (ORs) were estimated with logistic regression adjusting for province (bc, Ontario, Quebec), calendar time (biweekly epiweeks), and age group (12–49, 50–64, and 65+ years). VE derived as (1‐OR)*100%. All estimates are for those ≥ 12 years unless otherwise specified. Vaccinated participants are those who received fall 2024/25 vaccine ≥ 2 weeks prior to ARI onset. No participants in VE analyses received a spring 2025 booster dose prior to ARI onset. Time since vaccination refers to interval between receipt of last COVID‐19 vaccine and ARI onset among vaccinated participants. Analyses span epi‐weeks 44 to 18 but due to few vaccinated participants reaching 12+ weeks since vaccination during the first half of the season (*n* = 28 in epi‐weeks 44 to 4 versus *n* = 510 in epi‐weeks 5 to 18), we restricted the analysis of VE at 12+, 12–15 and 16+ weeks postvaccination to epi‐weeks 5 to 18. Minimal difference in VE (≤ 1% absolute) was observed when using Firth's logistic regression to address small sample size. For details on virological characterization, see Table S1.

At median time postvaccination of 10–11 weeks, VE against outpatient COVID‐19 was 54%, somewhat lower when restricted to ≥ 65‐year‐olds (40%) (Figure 1). Overall, VE decreased from 68% at 2–7 weeks to 27% at ≥ 12 weeks (median 17, IQR 14–20, range 12–28 weeks), notably negligible at ≥ 16 weeks (median 19, IQR 17–22 weeks) postvaccination. With comparable TSV (median 10–11 weeks), variant‐specific XEC versus non‐XEC estimates were similar (45% vs. 48%, respectively). In sensitivity analyses (Tables S2, S3), VE changed minimally (absolute difference) when including influenza test‐positive controls (−3% to −6%), excluding participants vaccinated ≤ 6 months before the fall vaccination campaign (+5% to +9%), adjusting for finer age strata (+1% to +2%) or sex and comorbidity (−1%), or using self‐reported versus PIR‐based vaccine status (−12% to +3%), between which agreement was moderate (Table S4) [8].

## 4 Discussion

The community‐based Canadian SPSN reports that the 2024/25 KP.2 mRNA vaccine halved the risk of COVID‐19 overall among ≥ 12‐year‐olds requiring outpatient medical visits for ARI. These findings of substantial risk reduction are especially meaningful given that by 5 years postpandemic the immunological landscape has dramatically changed, with VE now representing vaccine protection in a mostly SARS‐CoV‐2 experienced population and with most of the residual COVID‐19 healthcare burden now likely concentrated in outpatient settings [9]. As previously [10], we show VE was greatest within the first 2 months postvaccination, reducing the risk by about two‐thirds, but rapidly declining to negligible by the fifth month postvaccination.

With nearly three‐quarters of case viruses sequenced, we directly contextualize our VE findings within the mix of contributing variants. XEC and KP.3.1.1 variants were most common, each comprising about one‐third. In addition to the FLiRT/FLiQE substitutions T346R and Q493E relative to vaccine, both variants separately acquired N‐terminal domain substitutions and glycans of potential relevance to immune evasion [11, 12]. While both JN.1 and KP.2 vaccines elicit robust neutralizing antibody titres against all JN.1 sublineages (slightly higher for KP.2 vaccines), modest reductions have been reported against KP.3 and more so XEC/KP.3.1.1 [13, 14, 15, 16]. Absent serological thresholds for protection, the extent to which immunogenicity differences may explain suboptimal (albeit substantial) VE, even shortly postvaccination, remains unclear. Ideally, analysis of waning protection should simultaneously account for concomitant changes in variant contribution [10]. For the current work this includes early but brief predominance of KP.3.1.1 and stable but slightly increasing XEC contribution across the analysis period. Although sample size precluded finer TSV stratification, similar VE for XEC (45%) and non‐XEC (48%) variants at comparable median TSV suggests declining VE is not driven by shifts in variant contribution, as also reinforced by VE reported elsewhere against XEC and KP.3.1.1 [17, 18]. Additionally, while XEC increased across the SPSN season, it never comprised > 50% of variants, suggesting it was not a strong immune escape contender.

In 2024/25, findings from England, Denmark, and the US report sustained protection with stable VE after ≥ 20, ≥ 13 and a range of ~8–17 weeks postvaccination, respectively [18, 19, 20]. End‐of‐season analyses from the US IVY inpatient network show more stable VE against hospitalization, declining only after ≥ 25 weeks [17]. Of note, VE against COVID‐19 hospitalization has previously been shown higher and more durable than VE against outpatient illness [21]. Additional context, including the timing and contribution of overall SARS‐CoV‐2 activity and variant circulation, relative mix of vaccine products, participant ages and weighting within TSV strata, may also explain differences across jurisdictions. Whether earlier vaccination start elsewhere, more proximal to late‐summer SARS‐CoV‐2 activity, may contribute immunologically and/or methodologically to differences in VE patterns, also warrants consideration. Better understanding of the duration of protection is critical to optimal immunization programme timing, including prioritized target groups and the need for same‐season (e.g., spring) booster doses.

Most 2024/25 COVID‐19 VE studies have focused on older adults. Estimated VE among ≥ 65‐year‐olds in our study (40%) is comparable to older adult estimates from other test‐negative design studies, including against outpatient illness in the US VISION network (~35%) [19] and hospitalization in England (~35%–45%) [20], but lower than VE against outpatient illness in the US Veterans Affairs healthcare system (58%) [22], and in a European multicountry primary care analysis (66%) [23] or against hospitalization in a Danish cohort study (70%) [18]. As mentioned, other differences must be considered when comparing VE estimates, including methodological. For instance, the US Veterans Affairs analysis did not adjust for calendar time, despite indication of confounding, and reflects short‐term protection (median ~4 weeks postvaccination) from September–November [22]. Vaccine status ascertainment (e.g., self‐report, registry) and vaccine strain also varied elsewhere. In our study, we observed VE underestimation (up to 12% absolute) with self‐report, similar to findings elsewhere, likely reflecting nondifferential misclassification bias exacerbated by low vaccine coverage [24, 25]. The impact of variation in the chosen vaccine strain remains uncertain with a wide range of strain‐specific estimates (e.g., 40%–70% JN.1 VE against inpatient illness [19, 20] and 40%–60% KP.2 VE against outpatient illness [22]), but none directly comparing relative JN.1 versus KP.2 VE.

The main limitation of our study is the wide confidence intervals, reflecting low SARS‐CoV‐2 activity, vaccine coverage and VE point estimates, limiting statistical power and precision and precluding further stratified analyses. We uniquely provide genetic characterization of most contributing case viruses to help disentangle waning from differential variant‐specific contribution across the season; however, we cannot draw definitive conclusions. VE estimates for the 2024/25 season may also require consideration of the preceding summer SARS‐CoV‐2 peak and potential impact in lowering our VE estimates.

Overall, end‐of‐season findings from the Canadian SPSN add to the limited body of literature otherwise available for the postpandemic performance of seasonal COVID‐19 vaccination. We show COVID‐19 vaccination continues to provide added protection within a SARS‐CoV‐2‐experienced population, with the 2024/25 KP.2 vaccine reducing the risk of outpatient ARI due to SARS‐CoV‐2 by about half overall and by two‐thirds within 2 months postvaccination. As previously, however, we note protection against medically attended outpatient illness waned considerably by the fifth month postvaccination, with implications for pursuit of improved vaccine technologies and booster dose recommendations.

## Author Contributions

**Lea Separovic:** conceptualization, data curation, formal analysis, investigation, visualization, writing – original draft preparation, writing – review and editing. **Suzana Sabaiduc:** conceptualization, data curation, formal analysis, investigation, visualization, writing – original draft preparation, writing – review and editing. **Yuping Zhan:** data curation, formal analysis, investigation, project administration, writing – review and editing. **Samantha E. Kaweski:** data curation, investigation, project administration, writing – review and editing. **Sara Carazo:** data curation, funding acquisition, methodology, project administration, resources, supervision, validation, writing – review and editing. **Romy Olsa, Richard Mather, Christine Lacroix, Maan Hasso, Inès Levade, Isabelle Meunier, Agatha Jassem, Ruimin Gao and Nathalie Bastien:** data curation, funding acquisition, methodology, project administration, resources, supervision, writing – review and editing. **Katie Dover:** formal analysis, writing – review and editing. **Danuta M. Skowronski:** conceptualization, funding acquisition, investigation, methodology, project administration, resources, supervision, validation, writing – original draft preparation, writing – review and editing.

## Funding

This work was supported by funding provided by the bc Ministry of Health, Public Health Ontario, the Ministère de la santé et des services sociaux du Québec and the Public Health Agency of Canada (arrangement number 2324‐HQ‐000038).

## Disclosure

The views expressed herein do not necessarily represent the view of the Public Health Agency of Canada. Funders had no role in data analysis, interpretation or the decision to publish.

## Ethics Statement

This work was conducted as core surveillance and evaluation, with waiver of ethics review on that basis provided in each province. In British Columbia both the University of British Columbia Clinical and Behavioural Research Ethics Board (REB) waived review because such evaluations are considered within the core public health mandate of the bc Centre for Disease Control (BCCDC). In Ontario, the Public Health Ontario Ethics Review Board also determined the project did not require ongoing review as the activities are considered routine public health practice in fulfilment of Public Health Ontario's legislated mandate, and not research. In Quebec, such evaluations are similarly considered part of core public health surveillance for which reason the Centre Hospitalier Universitaire de Québec REB also provided separate waiver of review.

## Consent

Participating patients provided verbal consent.

## Conflicts of Interest

DMS is principal investigator on grants received to her institution from the Public Health Agency of Canada in support of this work. She has received grants from Pacific Public Health Foundation and Canadian Institutes of Health Research for unrelated work, also paid to her institution. SC reports funding from the Public Health Agency of Canada paid to her institution, but not pertaining to the current study. Other authors declare no conflicts of interest.

## Supporting information


**Table S1:** Lineage distribution of sequenced SARS‐CoV‐2 case viruses included in vaccine effectiveness analysis by province, Canadian Sentinel Practitioner Surveillance Network (SPSN), 27 October 2024 to 03 May 2025 (Weeks 44–18).
**Figure S1:** Epidemic curve of SARS‐CoV‐2 cases and controls, Canadian Sentinel Practitioner Surveillance Network (SPSN), 27 October 2024 to 03 May 2025 (Weeks 44–18).
**Figure S2:** Proportion of weekly SARS‐CoV‐2 case viruses by genetic lineage, Canadian Sentinel Practitioner Surveillance Network (SPSN), 27 October 2024 to 03 May 2025 (Weeks 44–18).
**Table S2:** Vaccine effectiveness against acute respiratory illness due to SARS‐CoV‐2, sensitivity analyses, Canadian Sentinel Practitioner Surveillance Network (SPSN), 27 October 2024 to 03 May 2025 (Weeks 44–18).
**Table S3:** Vaccine effectiveness against acute respiratory illness due to SARS‐CoV‐2, vaccine status per provincial immunization registry or self‐report, Canadian Sentinel Practitioner Surveillance Network (SPSN), 27 October 2024 to 19 April 2025 (Weeks 44–16).
**Table S4:** Vaccination status according to provincial immunization registry and self‐report, Canadian Sentinel Practitioner Surveillance Network (SPSN), 27 October 2024 to 19 April 2025 (Weeks 44–16).

## Data Availability

Aggregate epidemiological data are provided within the manuscript and Supporting Information. Any further sharing of data will be considered upon reasonable request to the corresponding author with appropriate review and aggregation, as required to comply with privacy and confidentiality. Sequencing data for SPSN SARS‐CoV‐2 viruses meeting provincial and/or national criteria for upload and their submitting and contributing laboratories can be found on GISAID using the Epi Set ID: EPI_SET_250903wr (https://doi.org/10.55876/gis8.250903wr).
