# Supplementary material for: Effectiveness of 2024/25 KP.2 Vaccine Against Outpatient COVID‐19 in Canada
Source: Influenza Other Respir Viruses. 2026 Mar 5;20(3):e70222. doi: 10.1111/irv.70222 (PMC12962023; doi:10.1111/irv.70222)
Supplement: Supplementary file 1 — Table S1: Lineage distribution of sequenced SARS‐CoV‐2 case viruses included in vaccine effectiveness analysis by province, Canadian Sentinel Practitioner Surveillance Network (SPSN), 27 October 2024 to 03 May 2025 (Weeks 44–18). Figure S1: Epidemic curve of SARS‐CoV‐2 cases and controls, Canadian Sentinel Practitioner Surveillance Network (SPSN), 27 October 2024 to 03 May 2025 (Weeks 44–18). Figure S2: Proportion of weekly SARS‐CoV‐2 case viruses by genetic lineage, Canadian Sentinel Practitioner Surveillance Network (SPSN), 27 October 2024 to 03 May 2025 (Weeks 44–18). Table S2: Vaccine effectiveness against acute respiratory illness due to SARS‐CoV‐2, sensitivity analyses, Canadian Sentinel Practitioner Surveillance Network (SPSN), 27 October 2024 to 03 May 2025 (Weeks 44–18). Table S3: Vaccine effectiveness against acute respiratory illness due to SARS‐CoV‐2, vaccine status per provincial immunization registry or self‐report, Canadian Sentinel Practitioner Surveillance Network (SPSN), 27 October 2024 to 19 April 2025 (Weeks 44–16). Table S4: Vaccination status according to provincial immunization registry and self‐report, Canadian Sentinel Practitioner Surveillance Network (SPSN), 27 October 2024 to 19 April 2025 (Weeks 44–16). [file IRV-20-e70222-s001.pdf]

Supplementary Materials

Table of Contents

Table S1. Lineage distribution of sequenced SARS-CoV-2 case viruses included in vaccine effectiveness analysis by province, Canadian Sentinel Practitioner Surveillance Network (SPSN), 27 October 2024 to 03 May 2025 (Weeks 44-18). 2

Figure S1. Epidemic curve of SARS-CoV-2 cases and controls, Canadian Sentinel Practitioner Surveillance Network (SPSN), 27 October 2024 to 03 May 2025 (Weeks 44-18). 3

Figure S2. Proportion of weekly SARS-CoV-2 case viruses by genetic lineage, Canadian Sentinel Practitioner Surveillance Network (SPSN), 27 October 2024 to 03 May 2025 (Weeks 44-18). 4

Table S2. Vaccine effectiveness against acute respiratory illness due to SARS-CoV-2, sensitivity analyses, Canadian Sentinel Practitioner Surveillance Network (SPSN), 27 October 2024 to 03 May 2025 (Weeks 44-18). 5

Table S3. Vaccine effectiveness against acute respiratory illness due to SARS-CoV-2, vaccine status per provincial immunization registry or self-report, Canadian Sentinel Practitioner Surveillance Network (SPSN), 27 October 2024 to 19 April 2025 (Weeks 44-16). 6

Table S4. Vaccination status according to provincial immunization registry and self-report, Canadian Sentinel Practitioner Surveillance Network (SPSN), 27 October 2024 to 19 April 2025 (Weeks 44-16). 6

References, Supplementary Material 7

Table S1. Lineage distribution of sequenced SARS-CoV-2 case viruses included in vaccine effectiveness analysis by province, Canadian Sentinel Practitioner Surveillance Network (SPSN), 27 October 2024 to 03 May 2025 (Weeks 44-18).

			BC<br>N = 49	Ontario<br>N = 277	Québec<br>N = 109	TOTAL<br>N = 435
**Case viruses successfully sequenced, n (% n/N)**			**42 (86%)**	**185 (67%)**	**85 (78%)**	**312 (72%)**
**Parental lineage[a, b]**		**Sub-lineages detected among SPSN case viruses[2, c]**				
	JN.1	KS.1.1, LB.1.3.1, LF.7.*, MV.1, NC.1.2.2, NL.*, PC.*, PY.2	2	14	8	24 (8%)
	LP.8.1	NY.*, PD.1, PR.2	6	23	8	37 (12%)
	KP.2	NM.2			1	1 (0%)
	KP.3	NP.1, PG.6	2	5	1	8 (3%)
	KP.3.1.1		3	26	12	41 (13%)
	MC	PA.1.4, PJ.1	7	43	27	77 (25%)
	XEC		18	68	24	110 (35%)
	Other	XEK.*, XEL.3, XEU, XFG, XFJ.1, NB.1.8.1[d], PQ.1.1[e]	4	6	4	14 (4%)

Abbreviations: BC, British Columbia; SPSN, Canadian Sentinel Practitioner Surveillance Network.

Whole genome sequencing of SPSN SARS-CoV-2 case viruses followed routine provincial or national laboratory protocols[2–7] and lineages assigned based on contemporary Pango nomenclature[1,8]. Data for SPSN SARS-CoV-2 viruses meeting provincial and/or national criteria for upload and their submitting and contributing laboratories can be found on GISAID using the Epi Set ID: EPI_SET_250903wr (https://doi.org/10.55876/gis8.250903wr).[9]

[a] All lineages include their descendants unless otherwise noted
[b] Lineages determined using pangolin version 4.3.1[1]
[c] Lineages suffixed with an asterisk also include their descendant lineages, unless otherwise noted
[d] Descendant of XDV viruses; two detected in weeks 17-18
[e] Descendant of NB.1.8.1 viruses; one detected in week 14

Figure S1. Epidemic curve of SARS-CoV-2 cases and controls, Canadian Sentinel Practitioner Surveillance Network (SPSN), 27 October 2024 to 03 May 2025 (Weeks 44-18).

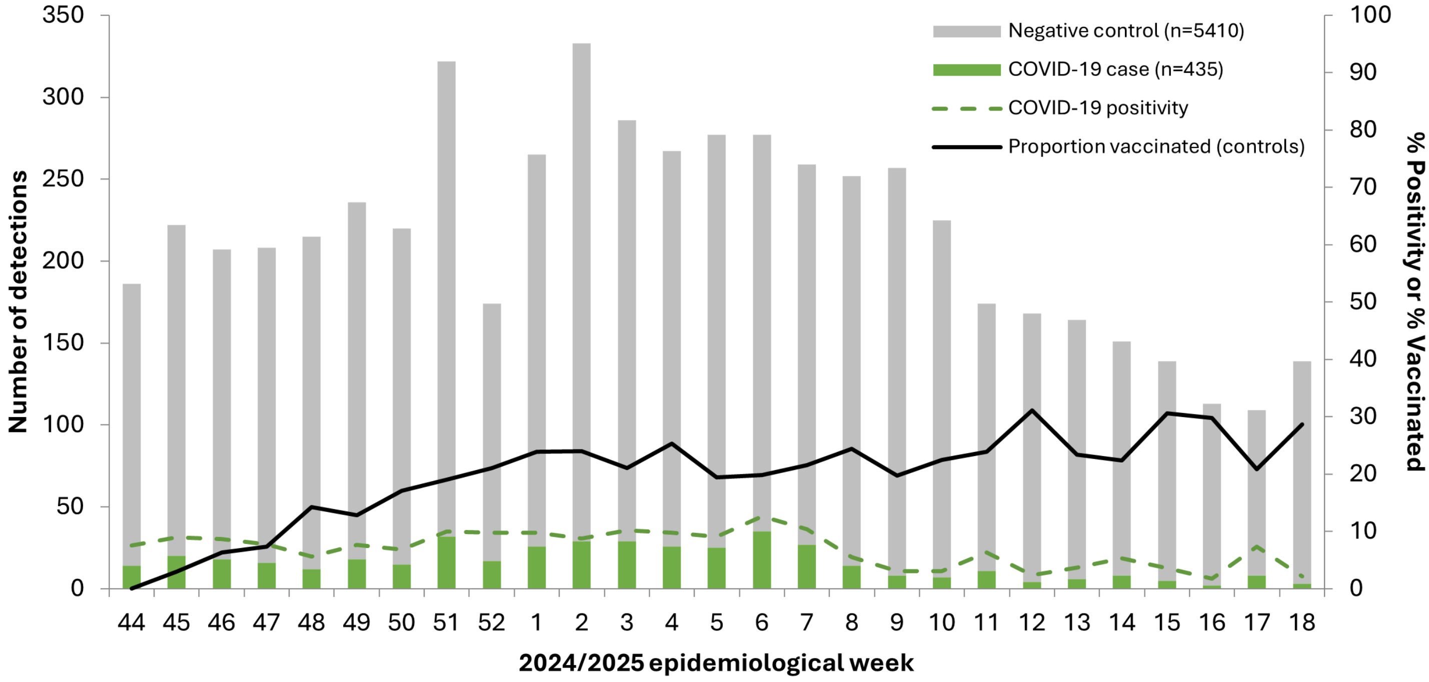

Displayed proportion vaccinated is based on those vaccinated ≥ 2 weeks before illness onset date.

Figure S2. Proportion of weekly SARS-CoV-2 case viruses by genetic lineage, Canadian Sentinel Practitioner Surveillance Network (SPSN), 27 October 2024 to 03 May 2025 (Weeks 44-18).

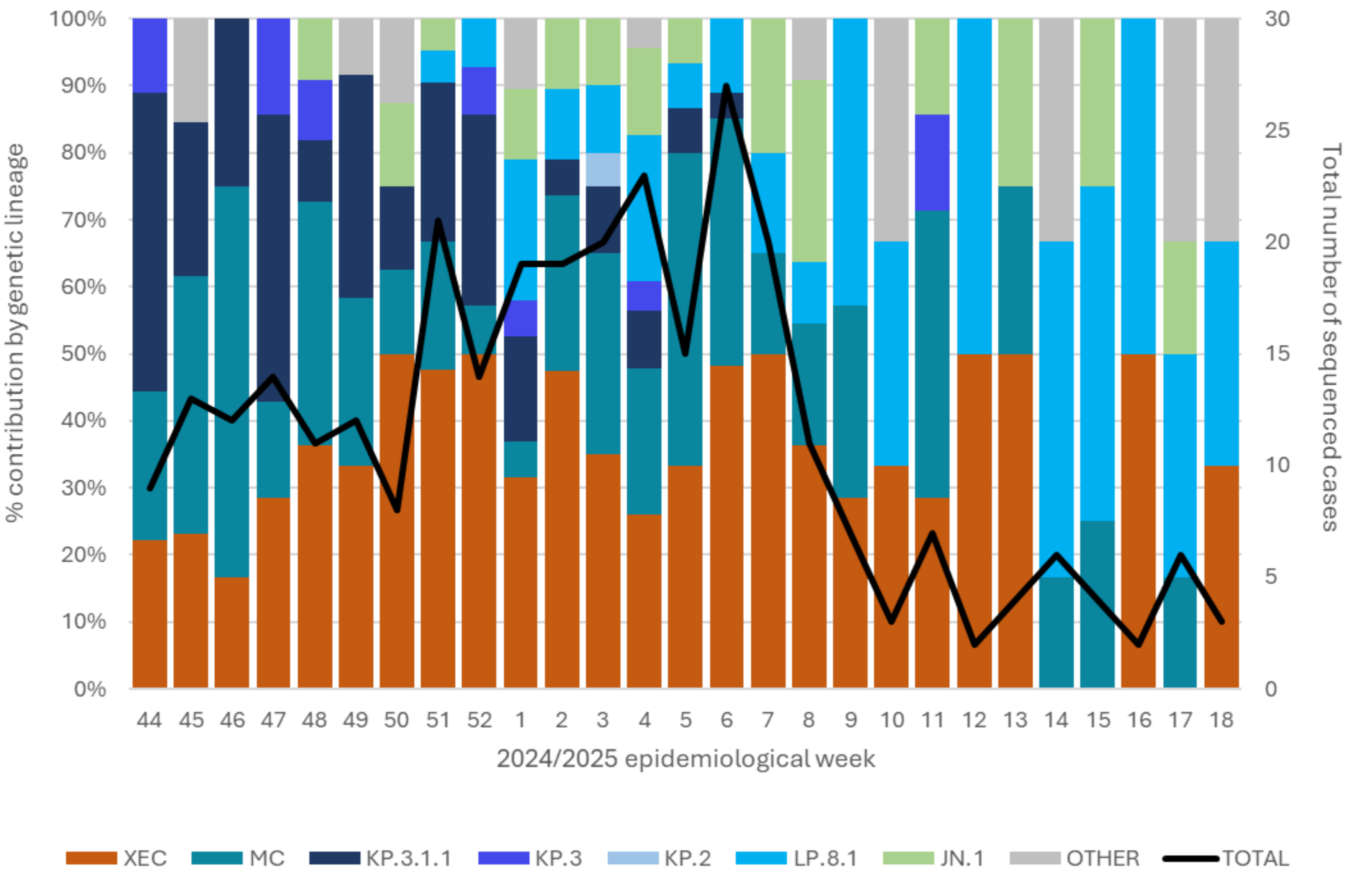

Among genetically sequenced SARS-CoV-2 case viruses that contributed to SPSN COVID-19 VE analysis (n=312), displayed is the percentage by epi-week that belonged to the specified lineage. All viral lineages displayed are descendants or recombinants of JN.1 lineages, except for recombinants denoted as 'Other'. See **Table S1** for more details.

Table S2. Vaccine effectiveness against acute respiratory illness due to SARS-CoV-2, sensitivity analyses, Canadian Sentinel Practitioner Surveillance Network (SPSN), 27 October 2024 to 03 May 2025 (Weeks 44-18).

	Total	Cases		Controls		Unadjusted OR[a]		Adjusted OR[a,b]		Adjusted VE[a,b]	
	N	n vac[c]/N	%	n vac[c]/N	%	OR	95% CI	OR	95% CI	VE	95% CI
**Primary analysis**											
≥ 12 years	5845	44/435	10	1053/5410	19	0.47	(0.33, 0.63)	0.46	(0.32, 0.64)	54	(36, 68)
≥ 65 years	1360	33/109	30	559/1251	45	0.54	(0.35, 0.81)	0.60	(0.38, 0.93)	40	(7, 62)
**Adjusted for additional age subgroups[d]**											
≥ 12 years	5845	44/435	10	1053/5410	19	0.47	(0.33, 0.63)	0.44	(0.31, 0.62)	56	(38, 69)
≥ 65 years	1360	33/109	30	559/1251	45	0.54	(0.35, 0.81)	0.59	(0.37, 0.91)	41	(9, 63)
**Adjusted for sex and comorbidity[e]**											
≥ 12 years	5394	42/414	10	931/4980	19	0.49	(0.35, 0.67)	0.47	(0.33, 0.66)	53	(34, 67)
≥ 65 years	1225	32/107	30	481/1118	43	0.57	(0.36, 0.86)	0.61	(0.39, 0.95)	39	(5, 61)
**Excluding those receiving a COVID-19 vaccine between April to September 2024**											
≥ 12 years	5598	33/418	8	896/5180	17	0.41	(0.28, 0.58)	0.41	(0.27, 0.59)	59	(41, 73)
≥ 65 years	1169	22/95	23	430/1074	40	0.45	(0.27, 0.73)	0.51	(0.30, 0.84)	49	(16, 70)
**Including influenza-positive SARS-CoV-2 controls**											
≥ 12 years	8101	44/435	10	1327/7666	17	0.54	(0.39, 0.73)	0.52	(0.36, 0.72)	48	(28, 64)
≥ 65 years	1667	33/109	30	688/1558	44	0.55	(0.36, 0.83)	0.63	(0.41, 0.98)	37	(2, 59)

Abbreviations: CI, confidence interval; OR, odds ratio; vac, vaccinated; VE, vaccine effectiveness.

[a] ORs compared test positivity between vaccinated and unvaccinated participants with logistic regression. VE derived as (1–OR) x 100.
[b] Adjusted for age group (12-49, 50-64, ≥65 years), province (BC, Ontario, Quebec), and calendar time (bi-weekly epi-weeks).
[c] Vaccination status based on provincial immunization registry. Participants vaccinated < 2 weeks before onset of symptoms or with unknown vaccination status or timing were excluded.
[d] Adjusted for additional age subgroups (12-24, 25-49, 50-64, 65-79, ≥80 years) relative to primary analysis (12-49, 50-64, ≥65 years). With finer age adjustment, variant-specific VE was similar (46% (5, 71) against XEC and 50% (21, 69) against non-XEC); stratifying by time since vaccination, VE was 69% (43, 85), 53% (19, 75), 48% (-10, 79), 31% (-15, 60), and 14% (-60, 57) after 2-7, 8-11, 12-15, ≥12, and ≥16 weeks post-vaccination, respectively.
[e] Adjusted for sex (male, female) and comorbidity status (yes, no). Participants with unknown sex or comorbidity status were excluded.

Table S3. Vaccine effectiveness against acute respiratory illness due to SARS-CoV-2, vaccine status per provincial immunization registry or self-report, Canadian Sentinel Practitioner Surveillance Network (SPSN), 27 October 2024 to 19 April 2025 (Weeks 44-16).

	Total	Cases		Controls		Unadjusted OR[a]		Adjusted OR[a,b]		Adjusted VE[a,b]	
	N	n vac[c]/N	%	n vac[c]/N	%	OR	95% CI	OR	95% CI	VE	95% CI
**Vaccination status per PIR[d]**											
≥ 12 years	5597	41/424	10	993/5173	19	0.45	(0.32, 0.62)	0.44	(0.31, 0.62)	56	(38, 69)
≥ 65 years	1284	30/106	28	516/1178	44	0.51	(0.32, 0.78)	0.56	(0.35, 0.87)	44	(13, 65)
**Vaccination status per self-report[d]**											
≥ 12 years	4995	55/391	14	1034/4604	22	0.57	(0.42, 0.75)	0.56	(0.41, 0.77)	44	(23, 59)
≥ 65 years	1130	30/100	30	473/1030	46	0.50	(0.32, 0.78)	0.53	(0.33, 0.83)	47	(17, 67)

Abbreviations: CI, confidence interval; OR, odds ratio; PIR, provincial immunization registry; vac, vaccinated; VE, vaccine effectiveness.

[a] ORs compared test positivity between vaccinated and unvaccinated participants with logistic regression. VE derived as (1–OR) x 100.
[b] Adjusted for age group (12-49, 50-64, ≥65 years), province (BC, Ontario, Quebec), and calendar time (bi-weekly epi-weeks).
[c] Vaccination status based on PIR or self-report as indicated. Participants vaccinated < 2 weeks before onset of symptoms or with unknown vaccination status or timing were excluded.
[d] Analyses restricted to epi-weeks 44-16 in order to exclude participants with most recent vaccine receipt potentially during Spring 2025 booster campaign, with rollout beginning in early April in SPSN provinces, given exact vaccination date unavailable for self-reported vaccination status.

Table S4. Vaccination status according to provincial immunization registry and self-report, Canadian Sentinel Practitioner Surveillance Network (SPSN), 27 October 2024 to 19 April 2025 (Weeks 44-16).

		Per PIR			Agreement Metrics		
		Vaccinated	Not vaccinated	Total	Kappa (95% CI)	Sensitivity (95% CI)	Specificity (95% CI)
**Overall**							
**Per self-report**	**Vaccinated**	796	265	1061	0.78 (0.75, 0.80)	90% (88, 92)	94% (93, 94)
	**Not vaccinated**	85	3816	3901			
	**Total**	881	4081	4962			
**Cases**							
**Per self-report**	**Vaccinated**	33	21	54	0.71 (0.61, 0.81)	94% (81, 99)	94% (91, 96)
	**Not vaccinated**	2	334	336			
	**Total**	35	355	390			
**Controls**							
**Per self-report**	**Vaccinated**	763	244	1007	0.78 (0.75, 0.81)	90% (88, 92)	93% (93, 94)
	**Not vaccinated**	83	3482	3565			
	**Total**	846	3726	4572			

Abbreviations: CI, confidence interval; PIR, provincial immunization registry.

Displayed are number of participants considered vaccinated or unvaccinated per PIR or self-report as indicated. Restricted to those included in both self-report and PIR-based analyses (n=4962). Participants vaccinated < 2 weeks before onset of symptoms or with unknown vaccination status or timing were excluded. Restricted to epi-weeks 44-16 in order to exclude participants with most recent vaccine receipt potentially during Spring 2025 booster campaign, with rollout beginning in early April in SPSN provinces, given exact vaccination date unavailable for self-reported vaccination status. Kappa statistics interpreted as follows: ≤0.20 = none; 0.21–0.40 = minimal; 0.40– 0.59 = weak, 0.60–0.79 = moderate, 0.80-0.90 = strong, and 0.91–1.00 = almost perfect agreement.[10]

References, Supplementary Material

1. O'Toole Á, Scher E, Underwood A, et al. Assignment of epidemiological lineages in an emerging pandemic using the pangolin tool. *Virus Evol*. 2021;7(2):veab064. doi:10.1093/ve/veab064

2. Freed N. "Midnight" SARS-CoV2 genome sequencing protocol using 1200bp amplicon primer set v2 and the Nanopore Rapid library kit v1. Published online April 8, 2023. Accessed August 13, 2025. https://www.protocols.io/view/34-midnight-34-sars-cov2-genome-sequencing-protoc-csjfwcjn

3. Hickman R, Nguyen J, Lee TD, et al. Rapid, High-Throughput, Cost Effective Whole Genome Sequencing of SARS-CoV-2 Using a Condensed One Hour Library Preparation of the Illumina DNA Prep Kit. Preprint posted online February 8, 2022. doi:10.1101/2022.02.07.22269672

4. ARTIC Network. 2023. SARS-CoV-2 version 5.3.2 Scheme Release. https://community.artic.network/t/sars-cov-2-version-5-3-2-scheme-release/462

5. Quick J. nCoV-2019 sequencing protocol v1. Published online January 22, 2020. Accessed August 13, 2025. https://www.protocols.io/view/ncov-2019-sequencing-protocol-bbmuik6w

6. Illumina COVIDSeq Test Instructions for Use. California: Illumina. Published online 2021. https://support.illumina.com/content/dam/illuminasupport/documents/documentation/chemistry_documentation/Illumina-COVIDSeq-Test/illumina-covidseq-testinstructions-for-use-canada-200004493-00.pdf

7. Bourgey M, Dali R, Eveleigh R, et al. GenPipes: an open-source framework for distributed and scalable genomic analyses. *GigaScience*. 2019;8(6):giz037. doi:10.1093/gigascience/giz037

8. Rambaut A, Holmes EC, O'Toole Á, et al. A dynamic nomenclature proposal for SARS-CoV-2 lineages to assist genomic epidemiology. *Nat Microbiol*. 2020;5(11):1403-1407. doi:10.1038/s41564-020-0770-5

9. Shu Y, McCauley J. GISAID: Global initiative on sharing all influenza data – from vision to reality. *Eurosurveillance*. 2017;22(13). doi:10.2807/1560-7917.ES.2017.22.13.30494

10. McHugh ML. Interrater reliability: the kappa statistic. *Biochem Medica*. Published online 2012:276-282. doi:10.11613/BM.2012.031
